# Kinesin-7 CENP-E in tumorigenesis: Chromosome instability, spindle assembly checkpoint, and applications

**DOI:** 10.3389/fmolb.2024.1366113

**Published:** 2024-03-15

**Authors:** Yu-Hao Yang, Ya-Lan Wei, Zhen-Yu She

**Affiliations:** ^1^ Department of Cell Biology and Genetics, The School of Basic Medical Sciences, Fujian Medical University, Fuzhou, China; ^2^ Key Laboratory of Stem Cell Engineering and Regenerative Medicine, Fujian Province University, Fuzhou, China; ^3^ Medical Research Center, Fujian Maternity and Child Health Hospital, Fuzhou, China; ^4^ College of Clinical Medicine for Obstetrics and Gynecology and Pediatrics, Fujian Medical University, Fuzhou, China

**Keywords:** kinesin, CENP-E, chromosome instability, cancer, aneuploidy, tumorigenesis

## Abstract

Kinesin motors are a large family of molecular motors that walk along microtubules to fulfill many roles in intracellular transport, microtubule organization, and chromosome alignment. Kinesin-7 CENP-E (Centromere protein E) is a chromosome scaffold-associated protein that is located in the corona layer of centromeres, which participates in kinetochore-microtubule attachment, chromosome alignment, and spindle assembly checkpoint. Over the past 3 decades, CENP-E has attracted great interest as a promising new mitotic target for cancer therapy and drug development. In this review, we describe expression patterns of CENP-E in multiple tumors and highlight the functions of CENP-E in cancer cell proliferation. We summarize recent advances in structural domains, roles, and functions of CENP-E in cell division. Notably, we describe the dual functions of CENP-E in inhibiting and promoting tumorigenesis. We summarize the mechanisms by which CENP-E affects tumorigenesis through chromosome instability and spindle assembly checkpoints. Finally, we overview and summarize the CENP-E-specific inhibitors, mechanisms of drug resistances and their applications.

## 1 Introduction

The human genome contains 45 different kinesins, which can be divided into 14 subfamilies according to the phylogenetic analysis and classification of the motor domain (Lawrence et al., 2004; Rath and Kozielski, 2012). Kinesin-7 CENP-E (Centromere protein E) was first discovered as a 312 kDa chromosome scaffold-associated protein, which is located at the centromere of chromosome at metaphase and then redistributed to the midbody at telophase (Yen et al., 1991; 1992). CENP-E is a plus-end-directed kinesin at the outer kinetochore plate and the fibrous corona of kinetochores (Thrower et al., 1996; Cooke et al., 1997; Kim et al., 2008). The 230-nm-long coiled-coil of CENP-E serves as a motile kinetochore tether for microtubule capture and chromosome alignment (Kim et al., 2008). CENP-E is required for chromosome congression, alignment, and metaphase-to-anaphase transition during cell division (McEwen et al., 2001; Putkey et al., 2002; Weaver et al., 2003).

The expression of the *CENP-E* gene varies in different types of cancer, and most of them are upregulated (Yuan et al., 2023). CENP-E acts both as an oncogene and as a tumor suppressor during tumorigenesis (Weaver et al., 2007). Low levels of chromosome instability can promote tumor initiation, while high levels of aneuploidy result in the suppression of tumor growth and eventually cell death (Weaver and Cleveland, 2007; Weaver et al., 2007; Silk et al., 2013). CENP-E participates in mitotic checkpoint and cell cycle control to prevent chromosome missegregation that leads to aneuploidy (Weaver et al., 2007).

Kinesin family motors are key regulators in cell division and have become potential targets for chemotherapeutic intervention and cancer treatment (Rath and Kozielski, 2012). Considering the relationship between CENP-E and tumorigenesis, CENP-E’s specific inhibitors (Henderson et al., 2009; Ding et al., 2010; Qian et al., 2010; Hirayama et al., 2013; Kung et al., 2014; Ohashi et al., 2015a; Yamane et al., 2019) have also been synthesized and verified. And only GSK923295 entered clinical phase I (Chung et al., 2012). In this review, we summarize molecular mechanisms of CENP-E and tumorigenesis from the perspectives of expression patterns, cell division, and aneuploidy. Furthermore, we highlight the applications of CENP-E inhibitors and drug resistance mechanisms in tumor research and treatment.

## 2 Structure and molecular kinetics of kinesin-7 CENP-E

**FIGURE 1:**
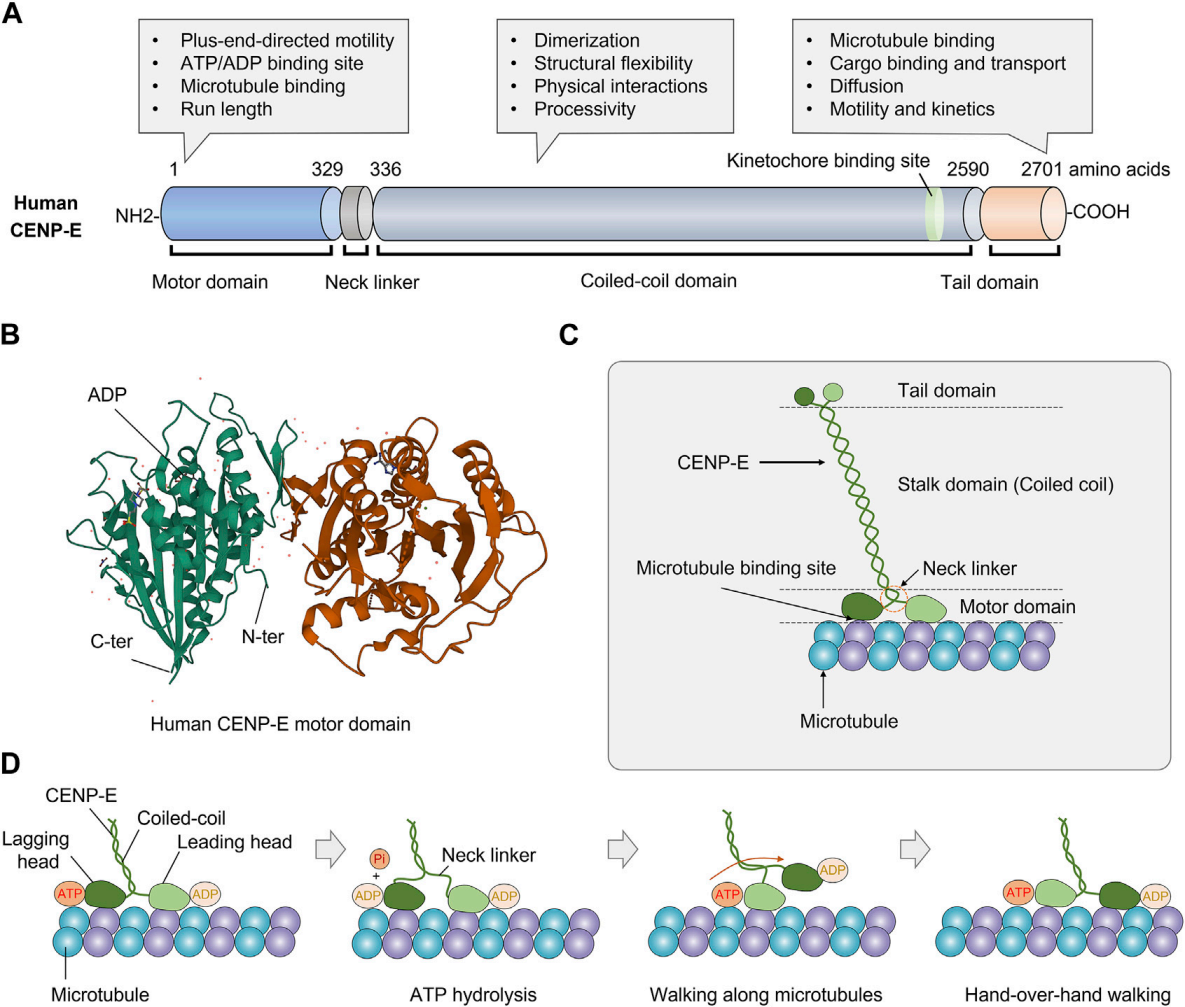
Structure and molecular kinetics of kinesin-7 CENP-E. **(A)** CENP-E is comprised of an N-terminal motor domain, a coiled-coil domain, and a C-terminal tail domain. The motor domain is required for plus-end-directed motility, ATP hydrolysis, microtubule binding, and run length. The coiled-coil domain is required for dimerization, structural flexibility, physical interactions with partner proteins, and processivity. The tail domain is essential for microtubule binding, cargo transport, diffusion along microtubules, motility, and kinetics. **(B)** Three-dimensional structure of human CENP-E motor domain (PDB database, No. 1T5C). The globular N-terminal motor domain contains the ATP/ADP binding site, which is essential for ATP hydrolysis and movement. **(C)** Schematic structure of kinesin-7 CENP-E. **(D)** The coordinated movement of CENP-E’s head domains along a microtubule. CENP-E is a processive motor that takes 8 nm steps along microtubules for each adenosine triphosphate hydrolyzed *via* a hand-over-hand mechanism.

CENP-E consists of an N-terminal motor domain, a central coiled-coil domain, and a C-terminal tail domain (Figure 1A). The N-terminal motor domain is highly conserved in diverse organisms (Craske et al., 2022) (Figure 1B). Unlike conventional kinesins, CENP-E has a 230-nm-long discontinuous coiled-coil, which forms different conformations *in vitro* and carries cargos in a compact configuration (Kim et al., 2008; Gudimchuk et al., 2018). The long coiled-coil domain mediates the motor functions and structural flexibility of CENP-E (Vitre et al., 2014; Taveras et al., 2019). The adjustable stalk configuration is required for physical interactions between CENP-E and spindle microtubules (Gudimchuk et al., 2018). The neck linker domain is responsible for the processivity of CENP-E motors (Hariharan and Hancock, 2009; Shastry and Hancock, 2011).

Human full-length CENP-E is predominantly inactive and becomes processive after microtubule binding (Craske et al., 2022). Most full-length CENP-E motors move at a slow velocity of 46.4 ± 1.88 nm/s (Craske et al., 2022). CENP-E motors show a higher run length and residency time than truncated CENP-E motors, which may be due to the non-motor microtubule-binding site at the C-terminal tail domain (Craske et al., 2022). Full-length CENP-E can walk to the microtubule plus end and maintain at the microtubule end for 20 s (Gudimchuk et al., 2013). In the tail domain, there is a kinetochore-targeting region (2055-2,450 amino acids) (Legal et al., 2020), a centrosome-targeting domain (2,260-2,608 amino acids), and a second microtubule-binding site (Gudimchuk et al., 2013; Ciossani et al., 2018) (Figure 1C). The C-terminal region (2091-2,358 amino acids) is essential for the recruitment of BubR1 at the kinetochores (Legal et al., 2020). The C-terminal domain also recruits the ROD-Zwilch-ZW10 (RZZ) complex, Spindly and Mad1 to the kinetochores (Weber et al., 2024). The tail domain is intrinsically disordered, which is a common structural feature of kinesins (Seeger et al., 2012). The tail domain truncated protein can diffuse along the microtubules with an average binding time of 0.5 s, suggesting a weak microtubule-binding affinity (Gudimchuk et al., 2013). Interestingly, the tail domain of CENP-E can inhibit the motility of CENP-E through the motor-tail interaction (Espeut et al., 2008). In different species, CENP-E is not conserved in the coiled-coil and tail domains, which are required to interact with partner proteins *in vivo*. This also contributes to the divergence in the kinetics and functions of CENP-E.

The crystal structure of the CENP-E motor domain associated with MgADP (PDB entry 1t5c) was reported (Garcia-Saez et al., 2004). The release of ADP is a rate-limiting step in the ATPase cycle of CENP-E (Sardar and Gilbert, 2012; Shibuya et al., 2021). The α0 helix is conserved in kinesin Eg5, however, these residues are disordered (Shibuya et al., 2021). The regions of α0 helix and Loop L1 are flexible in the motor domain of CENP-E (Shibuya et al., 2021) (Figure 1D). A recent study has revealed the crystal structure of CENP-E motor domain in complex with AMPPNP. And the helix α4 is required for the slow binding of CENP-E to microtubules (Shibuya et al., 2023). In the future, crystal structures of CENP-E in complex with specific inhibitors will help to elucidate the mechanisms of kinesin motors and the development of anticancer drugs.

## 3 Expression patterns of CENP-E in cancers: the contradiction between protective factor and oncogene

The high-level expression of CENP-E is closely related to its important functions during cell division. However, it is still unclear which factors regulate the high-level expression of CENP-E in the G_2_/M phase, which are fascinating questions that remain to be uncovered in the future. The upregulation of the expression level of CENP-E was involved in the tumorigenesis of various cancers. CENP-E is upregulated in human neuroblastoma (Balamuth et al., 2010), retinoblastoma (Shi et al., 2021), melanoma (Uzdensky et al., 2014), esophageal cancer (Zhu et al., 2019), lung adenocarcinoma (Shan et al., 2019), gliomas (Rahane et al., 2019; Xu et al., 2020), non-small cell lung cancer (Hao and Qu, 2019; Ma et al., 2019), basal-like subtype among breast cancer (Kung et al., 2014), chemotherapy-resistant epithelial ovarian cancer (Ju et al., 2009), and castration-resistant PCa (Liang et al., 2017). In addition, evaluation based on TCGA, GEPIA, and Oncomine databases has also revealed that the upregulation of the *CENP-E* gene in multiple tumor types, including colorectal cancer, cervical cancer, gastric cancer, breast cancer, lung cancer, and sarcoma (Shi et al., 2021). Expression patterns and key functions of CENP-E in multiple cancers are shown in Table 1.

**TABLE 1:** Expression patterns of CENP-E proteins in multiple cancers.

Cancer types	Type	Level	Functions of CENP-E	Loss-of-function phenotypes	References
Ovarian cancer	Gene	+	Stimulates cell proliferation, migration, and epithelial-mesenchymal transition	Obscure	Ju et al., 2009; Li et al., 2020
Prostate cancer	Protein	+	A novel biomarker and therapeutic target for the treatment of castration-resistant prostate cancer	Inhibition of cell growth	Liang et al. (2017)
Melanoma	Protein	+	Regulation of chromosome segregation; tumorigenesis	Obscure	Uzdensky et al. (2014)
Neuroblastoma	Gene	+	Stimulates tumor progression; a potential target.	Inhibition of cell growth; mitotic arrest	Balamuth et al. (2010)
Synovial sarcoma		+	A novel target for sarcoma diagnosis and prognosis	Obscure	Yao et al. (2021)
Retinoblastoma	Gene	+	A novel biomarker; correlated with the invasive behaviors	Obscure	Shi et al. (2021)
Medulloblastoma		+	A potential therapeutic target.	Activation of *TP53* or *TP73* and cell death signaling pathways	Iegiani et al. (2021)
Breast cancer	Gene	+	Associated with poor prognosis; increased JQ1 sensitivity	Obscure	Kung et al., 2014; Tian et al., 2021
Esophageal cancer	mRNA	+	Associated with poor overall survival of patients with adenocarcinoma; a novel biomarker and target.	Obscure	Zhu et al. (2019)
Lung adenocarcinoma	mRNA& Protein	+	Promotes the proliferation of LUAD cells	Inhibition of cell proliferation	Shan et al. (2019)
Non-small-cell lung cancer	mRNA	+	Involved in the occurrence and development of NSCLC.	Obscure	Ma et al., 2019; Hao and Qu, 2019
Glioma	mRNA	+	A novel biomarker and potential therapeutic target.	Obscure	Qi et al. (2020)
Hepatocellular carcinoma	mRNA&Protein	-	A tumor suppressor; associated with the prognosis of HCC; a potential biomarker or therapeutic target.	Aneuploidy	Liu et al., 2009; He et al., 2020; Liang et al., 2021
Acute lymphoblastic leukemia	mRNA	+/−	A tumor suppressor and oncogene	Obscure	Jiménez-Ávila et al. (2018)

**Note:** Lung adenocarcinoma, LUAD; Non-small-cell lung cancer, NSCLC; hepatocellular carcinoma, HCC; +, high expression level; -, low expression level.

In breast cancer cells, the *CENP-E* gene is overexpressed and associated with poor prognosis (Agarwal et al., 2009). The elevated expression levels of the *CENP-E* gene enhance the sensitivity of breast cancers to a drug, (+)—JQ1 (Tian et al., 2021). Moreover, the high expression of the *CENP-E* gene is associated with poor overall survival in patients with esophageal cancer and adenocarcinoma (Zhu et al., 2019). In retinoblastoma cell lines, the elevated expression of *CENP-E* positively correlates with tumor cell invasiveness (Shi et al., 2021), which suggests its potential role as a biomarker and drug target. *CENP-E* expression correlates with survival analyses in primary and recurrent synovial sarcomas, which may serve as a biomarker to indicate prognostic significance between metastasis and recurrence (Yao et al., 2021). Overexpression of CENP-E correlates with poor prognosis in the low-grade gliomas (Qi et al., 2020). And the expression level of CENP-E can be used as an indicator to evaluate the prognosis of esophageal squamous cell carcinoma (Shi et al., 2020) and osteosarcoma (Wang et al., 2020). These findings suggest that high expression level of CENP-E is closely related to poor prognosis and overall survival. Accumulating evidence has revealed that CENP-E is a candidate biomarker in cancer diagnosis and treatment.

siRNA or GSK923295-mediated CENP-E inhibition can activate *TP53* or *TP73* and cell death signaling pathways, suggesting that CENP-E may be a potential therapeutic target for medulloblastoma (Iegiani et al., 2021). Furthermore, proliferation of the aneuploid cells induced by CENP-E partial deletion using RNAi interference is counteracted by the p14^ARF^ tumor suppressor, indicating that p14^ARF^-p53 pathway is critical for preventing aneuploidy and chromosome instability in human cells (Veneziano et al., 2019). The interactions between CENP-E with kinesin-14 KIFC1 promote cell proliferation, migration, and epithelial-mesenchymal transition in ovarian cancers (Li et al., 2020). In castration-resistant PCa, genetic deletion or drug inhibition of CENP-E suppresses cell proliferation of prostate cancers (Liang et al., 2017). CENP-E is highly expressed in lung adenocarcinoma, and the downregulation of CENP-E is associated with the inhibition of the proliferation of lung cancer cells (Shan et al., 2019). In addition, the transcriptomic analysis revealed that *CENP-E* knockdown results in the downregulation of the pathways associated with G_2_/M checkpoint, mitotic spindle assembly checkpoint and the stress response in human primary fibroblasts (Cilluffo et al., 2021).

In contrast, the expression levels of *CENP-E* mRNAs and proteins are low in human hepatocellular carcinoma, and the low expression of CENP-E leads to aneuploidy in normal liver cell line LO2 cells (Liu et al., 2009). Furthermore, CENP-E functions as a tumor suppressor in human hepatocellular carcinoma (He et al., 2020). In addition, the *CENP-E* gene has also been shown to be associated with the prognosis of hepatocellular carcinoma cells and may be used as a biomarker or therapeutic target (Liang et al., 2021). But strangely, the *CENP-E* gene is highly expressed in LIHC (Yuan et al., 2023). It is still inconclusive as to why CENP-E is lowly expressed in hepatocellular carcinoma. Considering that only part of HCC shows consensus subtypes of chromosome instability (Lee et al., 2022), this difference may be caused by tumor heterogeneity. In contrast to most cancers, the low CENP-E expression in hepatocellular carcinoma is associated with increased cell proliferation, poor prognosis, and adverse clinical pathology (He et al., 2020). CENP-E is required for cell cycle control to prevent chromosome missegregation (Weaver et al., 2003). Reduction of CENP-E may produce aneuploidy (He et al., 2020), and chromosome instability, which is one of the subtypes of hepatocellular carcinoma (Lee et al., 2022). However, the specific roles of CENP-E in liver cancers as a tumor suppressor need to be further elucidated.

Furthermore, in acute lymphoblastic leukemia, there is a new alternative transcript of CENP-E (NAT-CENP-E) in patients, which is downregulated in 3/4 of the patients and upregulated in 1/4 of the patients (Jiménez-Ávila et al., 2018). In addition, XAB2 interacts with the promoter of CENP-E and transcriptionally activates the expression of CENP-E in HeLa cells (Hou et al., 2016). Taken together, these findings suggest that there are different factors or specific pathways for regulating the expression level of CENP-E in different types of tumors. However, the generality of the regulation of the CENP-E expression levels in different tumors needs to be further studied in the future.

## 4 Functions and mechanisms of kinesin-7 CENP-E in cell division

In mammalian cells, kinetochore fibers comprise 20-30 microtubules, which are essential for end-on attachment between the microtubule plus-ends and the kinetochore (McEwen et al., 1997). During mitosis, CENP-E proteins are enriched at unattached and misaligned kinetochores at prometaphase (Craske and Welburn, 2020), but detached from the aligned kinetochores at metaphase (Brown et al., 1994; Vitre et al., 2014). CENP-E proteins translocate from the kinetochores to the midbody at anaphase and telophase (Yen et al., 1991; Yao et al., 1997). Accumulating studies have revealed that BubR1 (Chan et al., 1999), Bub3 (Jiang et al., 2014; Li et al., 2016), Bub1 (Johnson et al., 2004), CENP-F, Mad1 (Akera et al., 2015), Astrin (Chung et al., 2016), SKAP (Huang et al., 2012) and small ubiquitin-related modifier (SUMO) proteins (Zhang et al., 2008; Wang and Dasso, 2009) are associated with kinetochore targeting of CENP-E proteins in mitosis. In turn, CENP-E recruits the kinetochore-associated proteins, including CLASP1 and CLASP2, to mediate microtubule turnover and poleward flux at the kinetochores (Maffini et al., 2009). CENP-E has also been shown to interact with CLASP through the C-terminal domain of CLASP (Gareil et al., 2023). Aurora A and B kinases phosphorylate CENP-E by releasing it from an autoinhibited state. At kinetochores, Aurora B phosphorylates CENP-E to inhibit its premature removal from kinetochores by dynein (Eibes et al., 2023).

**FIGURE 2:**
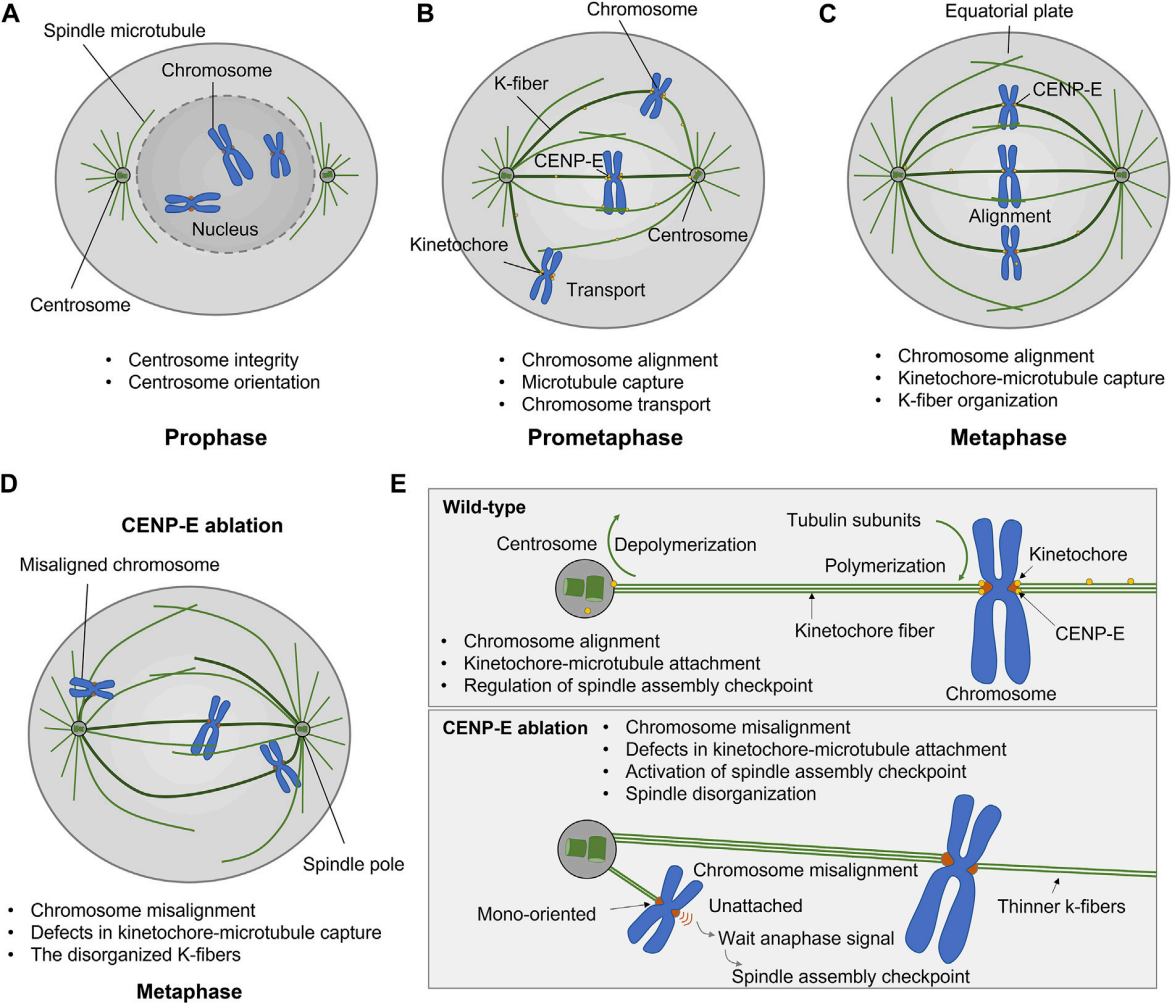
Kinesin-7 CENP-E is essential for kinetochore-microtubule attachment, chromosome alignment, and spindle assembly checkpoint in cell division. **(A–C)** CENP-E proteins are located at microtubules in prophase and accumulate at the kinetochores in prometaphase. CENP-E plays a key role in kinetochore-microtubule attachment and chromosome alignment during prometaphase and metaphase. **(D)** CENP-E ablation results in chromosome misalignment, spindle disorganization, and the activation of the spindle assembly checkpoint. **(E)** In wild-type cells, CENP-E proteins are essential for chromosome alignment, kinetochore-microtubule attachment, and the regulation of spindle assembly checkpoint. In the absence of CENP-E, the chromosomes are mono-oriented and misaligned, which further forms a wait anaphase signal and activates the spindle assembly checkpoint.

The antibody injection, dominant negative constructs, and genetic deletion of CENP-E both results in chromosome misalignment, which indicates that CENP-E is essential for chromosome congression and alignment (Schaar et al., 1997; Wood et al., 1997; Yao et al., 2000; McEwen et al., 2001; Maiato et al., 2017). CENP-E inhibition/deletion results in metaphase arrest with several mono-oriented chromosomes (McEwen et al., 2001), a delayed mitotic progression (Tanudji et al., 2004), and a decreased number of microtubules at kinetochore fibers (McEwen et al., 2001; Putkey et al., 2002; Weaver et al., 2003). Chromosome misalignment induced by CENP-E depletion is accompanied by mitotic spindle assembly defects, mitotic catastrophe, and severe spindle positioning defects (Tame et al., 2016; Iegiani et al., 2021; Owa and Dynlacht, 2021). In addition, CENP-E is related to microtubule flux in early mitosis, which is required for the conversion from lateral to end-on attachment and chromosome congression (Shrestha and Draviam, 2013; Barisic and Rajendraprasad, 2021) (Figure 2).

During prometaphase, CENP-E motors convert from a lateral mode to an end-on attachment mode in both the assembling and disassembling of microtubule plus-ends (Gudimchuk et al., 2013), which is required for chromosome movements and positioning. The combination of CENP-E and kinetochore protein Ndc80 supports lateral transport and microtubule wall-to-end transition at stabilized microtubules (Chakraborty et al., 2019). CENP-E binds to PRC1 through a conserved hydrophobic motif and promotes the antiparallel PRC1-crosslinked microtubules (Gluszek-Kustusz et al., 2023). During spindle assembly, PRC1-crosslinked microtubules undergo a network-to-bundles transition, and CENP-E promotes further microtubule bundling and kinetochore-mediated overlap formation (Matković et al., 2022).

CENP-E transports chromosomes to the spindle equator (Kapoor et al., 2006). CENP-E inhibition results in large chromosomes more vulnerable to defects in chromosome congression (Tovini and McClelland, 2019). CENP-E cooperates with chromokinesin KID and KIF4A to transport chromosomes toward the spindle equator along microtubules (Kapoor et al., 2006; Barisic and Rajendraprasad, 2021). The lateral kinetochore-microtubule attachment is mediated by CENP-E and dynein, which is required for chromosome congression (Maiato et al., 2017). During the initial poleward movement of peripheral chromosomes along astral microtubules, dynein is the dominant force counteracting the forces from CENP-E and chromokinesin in early mitosis (Barisic et al., 2014). During chromosome congression, CENP-E-mediated traction forces, in coordination with Kid-mediated forces on chromosome arms, are responsible for the loss of spindle pole integrity and multipolarity in CLASP1/2-depleted cells (Logarinho et al., 2012). Once the peripheral chromosomes reach the spindle pole, CENP-E becomes dominant over dynein and chromokinesin (Barisic and Maiato, 2016). There is a potential molecular switch between dynein and CENP-E activities on polar chromosomes (Barisic and Maiato, 2016) (Figure 2).

**FIGURE 3:**
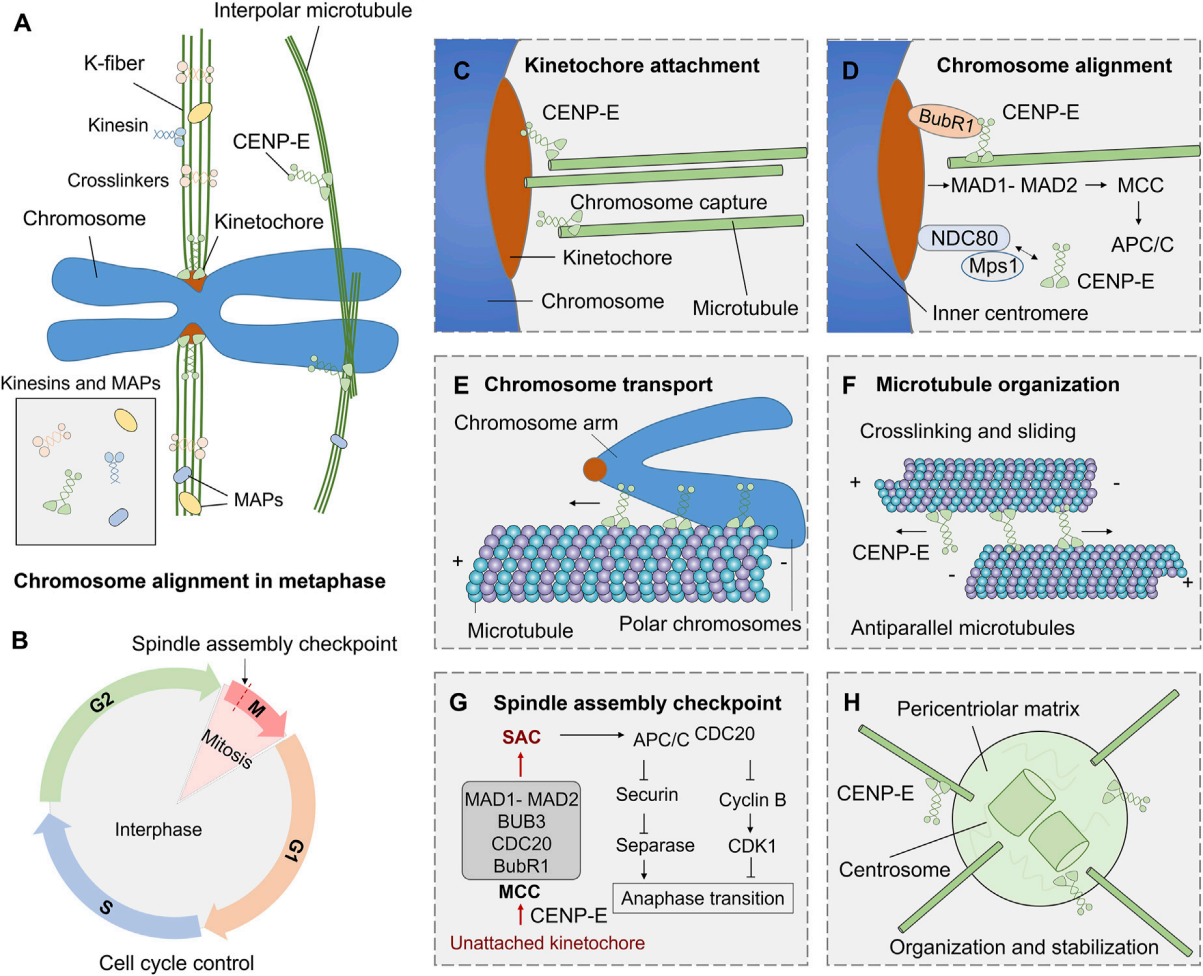
Functions and mechanisms of kinesin-7 CENP-E in cell division. **(A)** During mitosis, kinesins and microtubule-associated proteins (MAPs) are involved in microtubule crosslinking, kinetochore fiber assembly, and chromosome alignment. **(B)** The G_1_, S, G_2_, and M phases in the cell cycle are regulated by a complex cell cycle control system. **(C)** CENP-E associates with the plus ends of k-fibers and promotes kinetochore-microtubule attachment. **(D)** CENP-E interacts with BubR1, NDC80, Mps1, and kinetochore proteins to mediate chromosome alignment during metaphase. **(E)** CENP-E can transport polar chromosome arms along microtubules during prometaphase. **(F)** Both the motor and tail domains of CENP-E can bind to antiparallel microtubules and crosslink microtubules during spindle assembly. **(G)** The spindle assembly checkpoint pathway in mitosis. The unattached kinetochores on misaligned chromosomes can result in the formation of the mitotic checkpoint complex (MCC), including MAD1-MAD2, BUB3, CDC20, and BubR1 proteins, and then trigger the spindle assembly checkpoint. The checkpoint activates APC/C^CDC20^, inhibits Securin and separase, and then inhibits chromosome separation and regulates metaphase-to-anaphase transition. **(H)** CENP-E also mediates the organization of spindle poles and regulates centrosome organization and stabilization.

CENP-E is also essential for spindle assembly checkpoint in cell division (Chan et al., 1999; Abrieu et al., 2000; Guo et al., 2012). CENP-E interacts with multiple kinetochore proteins, including BubR1 (Legal et al., 2020), CENP-F (Chan et al., 1998), CLASP1 (Maffini et al., 2009), MAD1 (Akera et al., 2015). CENP-E is recruited by Bub1-Bub3 and BubR1-Bub3 complex at unattached kinetochores (Johnson et al., 2004). CENP-E is an activator of the BubR1 kinase, and CENP-E-dependent BubR1 autophosphorylation enhances chromosome alignment and the spindle assembly checkpoint (Mao et al., 2003; Guo et al., 2012). The basic C-terminal helix of BubR1 interacts with the minimal key acidic patch at the kinetochore-targeting domain of CENP-E to fulfill the recruitment of CENP-E to the kinetochores (Legal et al., 2020) (Figure 3). CENP-E is required for kinetochore recruitment of the corona’s building block consisting of ROD, Zwilch, ZW10, and the DD adaptor Spindly (RZZS). CENP-E proteins translocate to kinetochore through interactions with BubR1 and RZZS, and then mediate the kinetochore targeting of dynein-dynactin (Cmentowski et al., 2023). During fibrous corona formation, CENP-E interacts with Spindly and recruits RZZS to kinetochores through a farnesyl-dependent modification of its C-terminal kinetochore- and microtubule-binding domain (Wu et al., 2024).

## 5 Dual roles of CENP-E: synthesis requirement, chromosomal instability, or spindle assembly checkpoint

Aneuploidy was recognized as a characteristic of human cancer cells (Zasadil et al., 2016), and is usually accompanied by chromosome instability (McGranahan et al., 2012; Zasadil et al., 2013). Both aneuploidy and chromosome instability are the markers of poor prognosis in many tumor types (McGranahan et al., 2012; Zasadil et al., 2013). In yeast and murine cells, aneuploidy is associated with growth defects under optimal conditions (Torres et al., 2007; Williams et al., 2008). However, specific aneuploidy karyotypes can confer a growth advantage in response to certain stresses (Zasadil et al., 2016). In yeast, preexisting aneuploidy leads to accelerated growth in response to environmental stresses. Specific aneuploidy can evolve to overcome functional insufficiencies or adapt to environmental challenges (Rancati et al., 2008; Pavelka et al., 2010; Millet et al., 2015).

Tumorigenesis is associated with a lack of genomic integrity and genomic instability in cells, while chromosomal instability (CIN) and microsatellite instability (MIN) are also thought to be different mechanisms of cancer development (Yuen et al., 2005; Abbas et al., 2013; Pussila et al., 2018). It has been found that *CENP-E* heterozygosity cells can quickly induce aneuploidy *in vitro*, while aneuploidy can inhibit and promote tumorigenesis (Weaver et al., 2007), suggesting a dual role of *CENP-E* in tumorigenesis. *CENP-E* heterozygous deletion results in a low rate of chromosome segregation in liver cells, and causes high chromosomal instability and tumor suppression in the *Mad2* ^*+/−*^ mice (Silk et al., 2013). Furthermore, *CENP-E* heterozygous deletion also induces an increased level of aneuploidy and then leads to an elevated level of spontaneous lymphomas and lung tumors in the aged mice (Weaver et al., 2007; Silk et al., 2013).

A lower level of aneuploidy provides a growth advantage for tumorigenesis and promotes tumorigenesis (Weaver and Cleveland, 2006), while a higher level of instability inhibits its growth (Weaver et al., 2007). But whether it is “promoting cancer” or “inhibiting cancer” depends on the cell type and whether there is additional genetic damage. The functional defects of CENP-E can induce CIN in different tissues. For example, in human soft tissue sarcoma, loss of NF-kB activating protein (NKAP) leads to CENP-E mislocalization, which in turn leads to chromosomal missegregation and aneuploidy dysregulation that ultimately promotes tumorigenesis (Li et al., 2016). Meanwhile, depletion of CENP-E in epithelial tissues unable to activate the apoptosis has also been observed to induce significant levels of aneuploidy and drive tumor-like growth (Clemente-Ruiz et al., 2014). The rate of chromosome missegregation based on CENP-E has also been found to have such a dual effect and is synchronized with the similar effect of aneuploidy (Silk et al., 2013). But when compared with the expression levels of CENP-E in tumors, CENP-E is more likely to promote tumor growth, and may only play a role as a tumor suppressor in liver cancer and acute lymphoblastic leukemia. The ability of CENP-E to inhibit and promote cancer in acute lymphoblastic leukemia may be the result of alternative splicing of *CENP-E* transcripts of mRNA (Jiménez-Ávila et al., 2018). This dual mechanism may mean that increasing the rate of chromosome missegregation can be used as a successful chemotherapy strategy (Funk et al., 2016).

Cancer cells usually harbor chromosome abnormalities and abnormal ploidy, which can result in specific constraints on the evolution of genetic changes (Gordon et al., 2012; Podgornaia and Laub, 2015; Bakhoum and Cantley, 2018). In uveal melanomas, *CENP-E* is a significantly mutated gene. *CENP-E* mutations are correlated with a higher percentage of chromosome copy number alterations (Johansson et al., 2020), but the underlying mechanisms are obscure. Furthermore, in follow-up studies, high levels of chromosomal instability based on *CENP-E* heterozygous have been shown to not inhibit tumor cell initiation, but inhibit subsequent cell growth (Zasadil et al., 2016).

**FIGURE 4:**
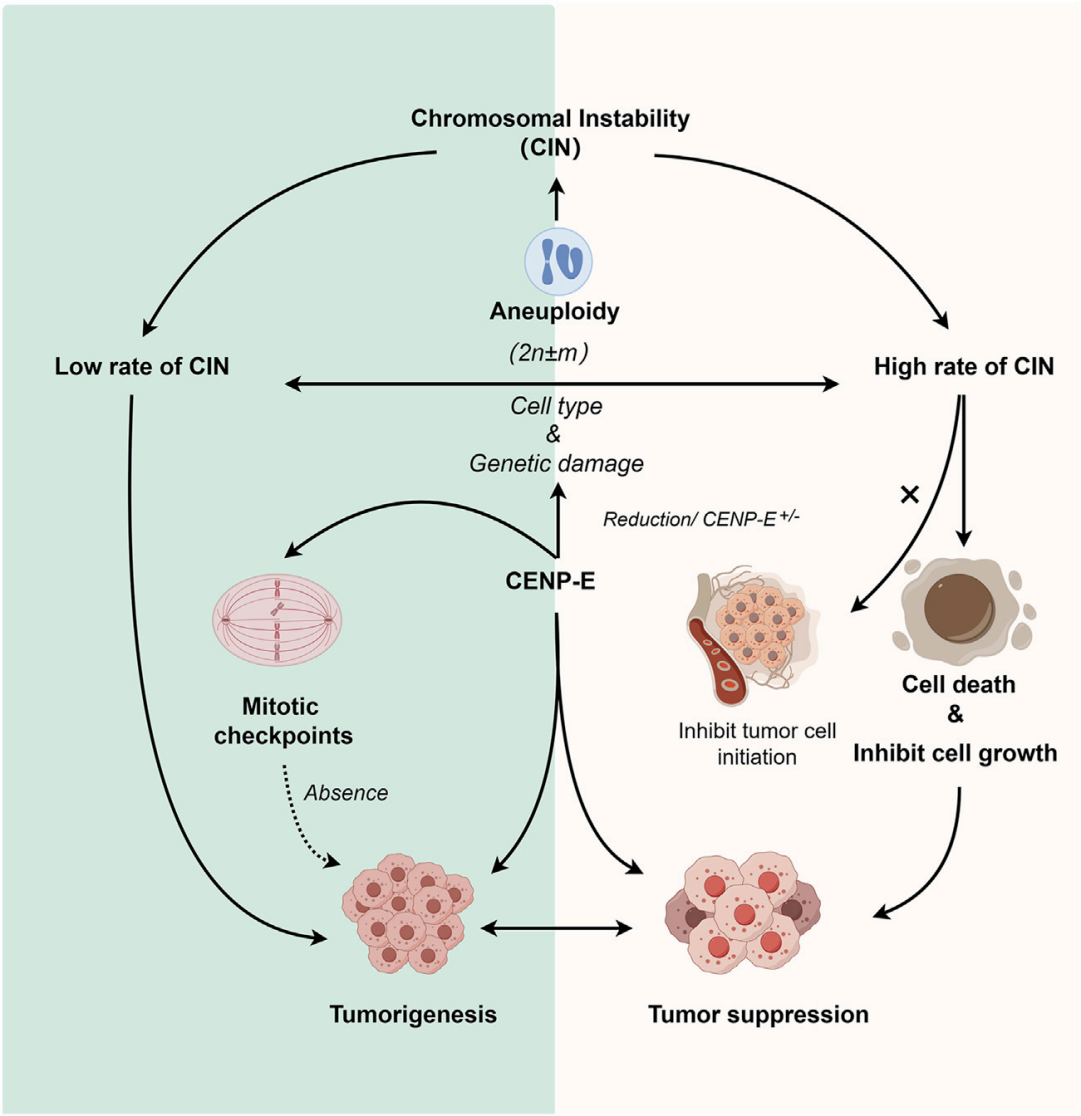
Dual roles of CENP-E in tumorigenesis. Reduction of CENP-E or *CENP-E* ^*+/−*^ can induce the occurrence of aneuploidy, and aneuploidy is highly related to chromosomal instability (CIN). *CENP-E* ^*+/−*^ can induce high or low rates of chromosomal instability, which depends on the cell type and genetic damage. A low rate of chromosomal instability can promote tumorigenesis, while a high rate of chromosomal instability will lead to cell death or tumor cell growth inhibition (but not tumor cell initiation). CENP-E is also involved in mitotic checkpoint, and the loss of mitotic checkpoint can also lead to tumorigenesis, suggesting another pathway for CENP-E-induced tumorigenesis.

CENP-E is a crucial regulator in mitotic checkpoint, and the absence of mitotic checkpoint will lead to tumorigenesis (Kops et al., 2005). Cells with a reduced level of CENP-E can enter the anaphase in the presence of misaligned chromosomes due to the weakened mitotic checkpoint. This results in a low rate of chromosome instability (Weaver et al., 2003; Weaver et al., 2007). In primary mouse embryonic fibroblasts with reduced levels of CENP-E, polar chromosomes are missegregated in 25% of divisions (Weaver et al., 2003). In head and neck cancers, polar chromosomes produced by decreased levels of CENP-E proteins lead to the occurrence of chromosomal instability, which may lead to tumorigenesis (Cosper et al., 2023). Resveratrol exhibits a biphasic effect on chromosomal instability, low doses of Resveratrol may reduce spontaneous chromosome instability, while high doses may induce chromosomal instability in human normal cells (Guo et al., 2018). Cells with a reduced level of CENP-E can enter the anaphase in the presence of misaligned chromosomes due to the weakened mitotic checkpoint, which further suggest that the dual effect occurs through the comprehensive regulation of the spindle assembly checkpoint pathway (Figure 4).

## 6 Discovery and applications of CENP-E inhibitors in cancer treatment and therapy

**FIGURE 5:**
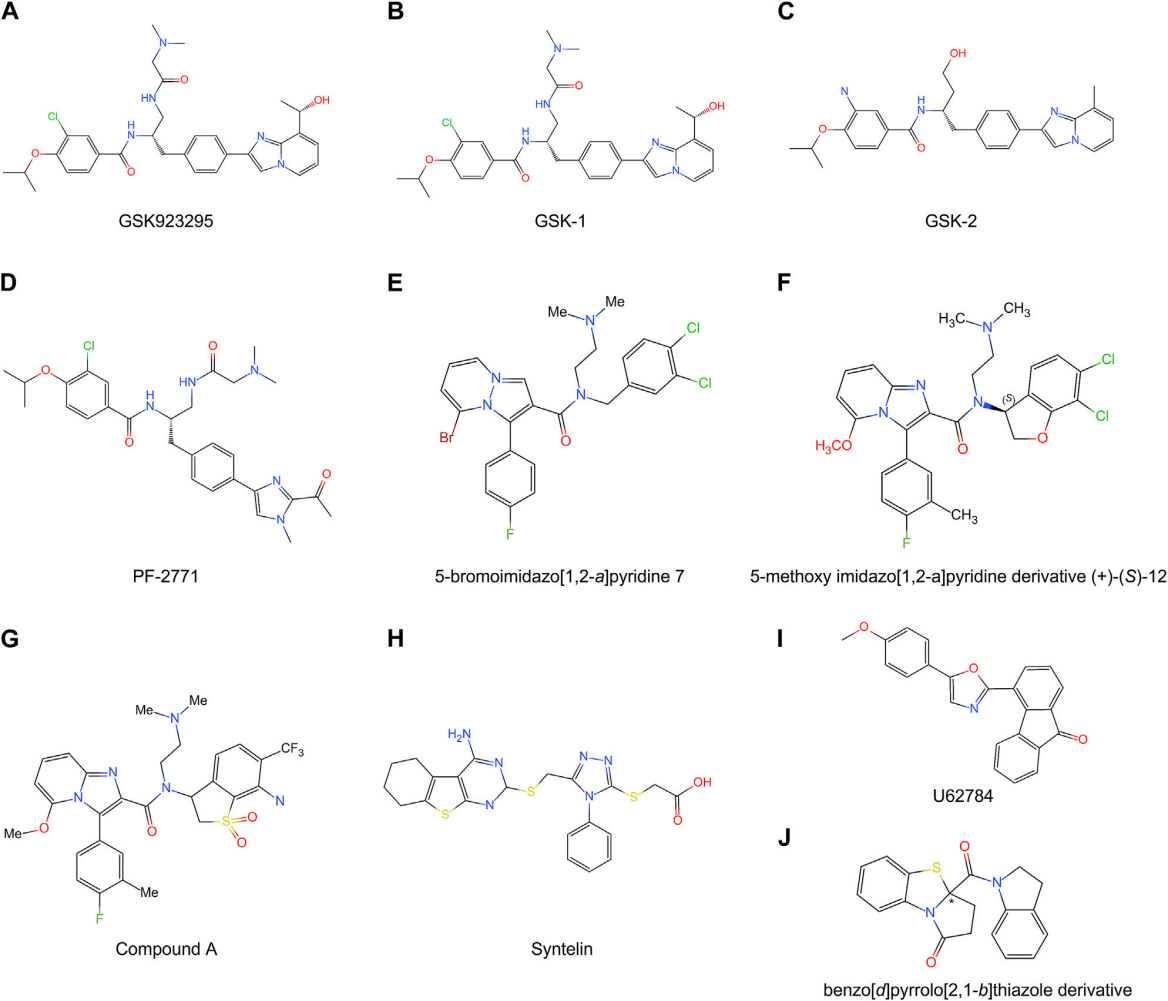
Chemical structures of multiple CENP-E inhibitors. **(A)** GSK923295; **(B)** GSK-1; **(C)** GSK-2; **(D)** PF-2771; **(E)** 5-bromoimidazo [1,2-*a*]pyridine 7; **(F)** 5-methoxy imidazo [1,2-a]pyridine derivative (+)-(*S*)-12; **(G)** Compound A; **(H)** Syntelin; **(I)** U62784; **(J)** benzo [*d*]pyrrolo [2,1-*b*]thiazole derivative.

Cancer cells are a population of cells with the ability to proliferate. The cytotoxic agents of cancers can be divided into four main kinds, including DNA alkylating agents, topoisomerases I and II inhibitors, antimetabolite agents, and microtubule targeting agents (Calligaris and Lafitte, 2011; Tcherniuk et al., 2011). Microtubule targeting agents can disrupt spindle assembly and microtubule dynamics, which are excellent cancer chemotherapeutic targets (Jordan and Wilson, 2004). Paclitaxel and the *Vinca* alkaloids are the most successful microtubule-target chemotherapeutic drugs that suppress microtubule dynamics and chromosome alignment, which results in mitotic arrest and apoptosis (Jordan and Wilson, 2004; Maiato et al., 2017). However, considering the neurotoxicity, neutropenia, and chemical resistance of microtubule-target agents, the discovery of novel anti-mitotic agents that do not disrupt microtubules is an emerging trend in cancer treatment (Jordan and Wilson, 2004). To date, seven kinds of CENP-E inhibitors have been found and synthesized (Table 2), which mainly inhibit chromosome alignment, and induce cell cycle arrest and eventually cell death. These CENP-E inhibitors might be novel anti-mitotic agents for cancer treatment (Figure 5).

**TABLE 2:** Summaries and characterizations of the binding sites, mechanisms, and phenotypes of CENP-E inhibitors.

Inhibitors	Inhibitor binding site/mechanisms	Phenotypes	References
GSK923295	Between helices α2 and α3, near loop 5 of the motor domain. Inhibits microtubule-stimulated ATPase of CENP-E	Chromosome misalignment, cell cycle arrest, apoptosis, cancer cell growth inhibition	Wood et al., 2010; Qian et al., 2010
GSK-1	Between helices α2 and α3, near loop 5 of the motor domain	Chromosome misalignment, cell cycle arrest	Wood et al. (2010)
GSK-2	Between helices α2 and α3, near loop 5 of the motor domain	Chromosome misalignment, cell cycle arrest	Wood et al. (2010)
PF-2771	Inhibits CENP-E’s motor activity	Chromosome instability, DNA damage, apoptosis, tumor growth regression	Kung et al. (2014)
Syntelin	Bind to different sites, inhibits CENP-E motility	Chromosome misalignment, disorganized central spindle, metaphase arrest, and apoptosis	Ding et al. (2010)
Imidazo [1,2-*a*]pyridine	Binds to the loop 5 binding site at the motor domain	Chromosome misalignment, mitotic arrest, tumor growth inhibition	Hirayama et al., 2013, 2015
Compound A	Inhibit the ATPase activity of the motor domain	Chromosome misalignment, mitotic arrest, SAC activation	Ohashi et al., 2015a, b
UA62784^*^	Inhibit microtubule-associated ATPase activity; stimulate microtubule depolymerization	Cell cycle arrest and apoptosis in pancreatic carcinoma	Henderson et al., 2009; Tcherniuk et al., 2011
Benzo [*d*]pyrrolo [2,1-*b*]thiazole derivatives	Inhibit the microtubule-stimulated ATPase activity of the motor domain	Cell cycle arrest, apoptosis, and the inhibition of cell proliferation	Yamane et al. (2019)

*: UA62784 has been proved that it is not an inhibitor of CENP-E (Henderson et al., 2009; Tcherniuk et al., 2011).

### 6.1 GSK923295 and its derivatives

GSK923295 is an allosteric and uncompetitive CENP-E inhibitor of both ATP and microtubules (Qian et al., 2010), which specifically binds to the motor domain of CENP-E and inhibits CENP-E microtubule-stimulated ATPase activity with a Ki of 3.2 ± 0.2 nM (Wood et al., 2010) (Figure 5A). Site-directed mutagenesis reveals that GSK923295 interacts with Ile182 and Thr183, and interacts with CENP-E as sandwiched between helices α2 and α3 and near loop 5 (Wood et al., 2010). GSK923295 inhibits the release of inorganic phosphate and locks the motor domain of CENP-E at microtubules (Wood et al., 2010). In cultured cancer cells and mouse tumor xenografts, CENP-E inhibition by GSK923295 leads to chromosome misalignment, cell apoptosis, and tumor regression (Qian et al., 2010; Wood et al., 2010).

In addition, GSK-1 (Figure 5B) shows an ATP competitive behavior, which is different from the ATP uncompetitive behavior of GSK923295 (Wood et al., 2010). These differences are caused by chemical modifications of the carbon extension of a sidechain (Qian et al., 2010). Due to this small difference, GSK-1 may bind to the sites overlapping with the binding site of GSK923295. GSK-2 (Figure 5C) is a closely related inhibitor of GSK923295 (Wood et al., 2010). GSK923295 and GSK-2 result in cell cycle arrest in mitosis and tumor regression *in vivo* (Wood et al., 2010). The examination of the growth inhibitory activity of GSK923295 in 237 tumor cell lines shows that the GI_50_ values of 212 cell lines are less than 100 nM (Wood et al., 2010). Further studies have revealed that GSK923295 shows antitumor activity in neuroblastoma cells (Balamuth et al., 2010), Ewing sarcoma, rhabdoid, rhabdomyosarcoma xenografts (Lock et al., 2012), and hepatocellular carcinoma (Tang et al., 2019). As the only CENP-E-specific inhibitor entering clinical trials, the synthesis process, modification method, and target site of GSK923295 can provide a reference for the subsequent development of new CENP-E inhibitors.

The Phase I, first-in-human study has revealed that the maximum-tolerated dose of GSK923295 is 190 mg/m^2^ and examined the safety, dose-proportional pharmacokinetics, and preliminary clinical activity of GSK923295 (Chung et al., 2011). Among all 39 patients, 33% of patients had a response of stable disease, 54% had progressive disease, and most patients had mild adverse events, including fatigue, gastrointestinal toxicities of diarrhea, nausea, vomiting, and anemia (Chung et al., 2012). GSK923295 can inhibit CENP-E with high penetrance and at a low effective dose in medulloblastoma cells (Iegiani et al., 2021). The combination of GSK923295 and pharmacologic inhibitors of mitogen-activated ERK kinase (MEK1/2) shows a significant synergistic growth inhibition on neuroblastoma, lung, pancreatic, and colon carcinoma cell lines, which further results in mitotic arrest and apoptosis (Mayes et al., 2013). GSK923295 significantly inhibits the proliferation of tetraploid cells compared with diploids, suggesting superior generality of CENP-E-targeted tetraploidy inhibition (Yoshizawa et al., 2023). These findings indicate that in cancer treatment, exploring the combination of GSK923295 with other antitumor drugs might improve its clinical application.

Furthermore, the in-depth exploration of the off-target effects of GSK923295, the half-life of the drug *in vivo*, pharmacokinetics, and tumor targeting efficiency can improve its clinical effects. The binding site of GSK923295 to CENP-E can be further clarified by site-directed mutagenesis of CENP-E protein, CRISPR-Cas9 gene editing technology, and protein-drug crystal structure analysis in the future. By modifying the chemical moiety of GSK923295, its tissue and cell penetration ability, solubility and half-life can be further improved, which can enhance its effects and clinical applications.

### 6.2 PF-2771

PF-2771 is a non-competitive and selective inhibitor of CENP-E, which specifically suppresses cell growth of basal breast cancer cells, resulting in chromosome instability, increased phosphor-HH3-Ser10 levels, and tumor growth regression (Kung et al., 2014) (Figure 5D). PF-2771 inhibits the motor activity of CENP-E with an IC_50_ of 16.1 ± 1.2 nM (Kung et al., 2014). The treatment of PF-2771 results in elevated expression of BubR1, Aurora B, securin, and Cyclin B, increased DNA damage, and apoptosis (Kung et al., 2014). PF-2771, similar to GSK923295, induces a high effect on chromosome instability and loss of human artificial chromosomes (Kim et al., 2016). PF2771 and GSK923295, along with paclitaxel, olaparib, and talazoparib (Lee et al., 2016) can be candidates for cancer therapy when chromosome instability is a therapeutic target.

To date, researchers have claimed that a variety of CENP-E inhibitors induce “high chromosome instability” (Kim et al., 2016). However, few studies concerning the effectiveness and differences of CENP-E inhibitors in chromosome instability. In addition, the binding sites and inhibition modes of these CENP-E inhibitors, such as PF-2771, remain largely unknown. Site-directed mutagenesis and *in vitro* experiments, as well as the analyses of the structures of the drug-bound CENP-E proteins, would help to discover the specific binding sites and mechanisms of the inhibitors in the future. Furthermore, cross-detection of the responses of different drug-resistant cell lines to different CENP-E inhibitors can verify whether the binding sites of the inhibitors are consistent.

### 6.3 Imidazo [1,2-*a*]pyridine scaffold derivatives and compound A

The imidazo [1,2-*a*]pyridine scaffold derivatives are another inhibitors of CENP-E, including 5-bromoinidazo [1,2-*a*]pyridine 7 (Hirayama et al., 2013) (Figure 5E) and 5-methoxy imidazo [1,2-*a*]pyridine derivative (+)-(*S*)-12 (Hirayama et al., 2015) (Figure 5F). Based on a fused bicyclic compound, 4, 5-dihydrothieno [3, 4-*c*]pyridine-6-carboxamide 1a, researchers synthesized a new 5-bromoimidazo [1,2-*a*]pyridine 7, which shows the potent in CENP-E inhibition with an IC_50_ at 50 nM and binds to the loop 5 binding sites at the motor domain of CENP-E (Hirayama et al., 2013). By site-direct mutagenesis and electrostatic potential map analyses, the modification of imidazo [1,2-*a*]pyridine scaffold led to the discovery of 5-methoxy imidazo [1,2-*a*]pyridine derivative (+)-(*S*)-12, which inhibits CENP-E with an IC_50_ at 3.6 nM, suppresses cell growth of HeLa cells at GI_50_ at 130 nM and shows antitumor activities in a Colo205 xenograft model (Hirayama et al., 2015). The docking model suggests that the imidazo [1,2-*a*]pyridine inhibitors interact with Pro107, Ile182, and loop 5 at CENP-E (Hirayama et al., 2015).

Based on imidazo [1,2-*a*]pyridine scaffold derivatives, researchers further synthesized 6-cyano-7-trifluoromethyl-2,3-dihydro-1-benzothiophene 1,1-dioxide derivative (+)-5d (Compound A) (Hirayama et al., 2015) (Figure 5G). Compound A is a time-dependent CENP-E inhibitor with ATP competitive behavior, which effectively inhibits the motor activity of CENP-E (Ohashi et al., 2015a). Compound A induces chromosome misalignment, prolonged mitotic arrest, and antiproliferation in multiple cancer cell lines (Ohashi et al., 2015a). Furthermore, Compound A shows strong anti-tumor activity in the COLO205 xenograft nude mouse tumor model and induces the activation of spindle assembly checkpoint in a variety of tumor cell lines (Ohashi et al., 2015a). In addition, CENP-E inhibition by Compound A causes chromosome missegregation, the *p53* gene-mediated post-mitotic apoptosis, which finally leads to proteotoxic stress and DNA damage in spindle assembly checkpoint-attenuated cells. However, polyploidy caused by Eg5 inhibition using Ispinesib under the same conditions does not result in proteotoxic stress and DNA damage (Ohashi et al., 2015b).

### 6.4 Syntelin

Syntelin is a novel class of CENP-E inhibitor, which inhibits the motility of CENP-E in a dose-dependent manner with an IC_50_ value of 160 nM (Ding et al., 2010) (Figure 5H). Compared with GSK923295, syntelin interacts with different regions outside the GSK923295s binding site and induces the inhibition of GSK923295-resistant cells (Ding et al., 2010). In HeLa cells, syntelin treatment results in misaligned chromosomes, reduced centromere stretches (Ding et al., 2010), and the disruption of the PRC1-organized central spindle (Liu et al., 2020a). The inhibition of CENP-E by syntelin causes metaphase arrest of HeLa cells and a syntelic attachment of spindle on chromosomes (Ding et al., 2010; Liu et al., 2020b). Syntelin treatment in triple-negative breast cancer, such as MDA-MB-231 cells, results in chromosome misalignment, the suppression of cell proliferation, and Bax-elicited apoptosis (Mullen et al., 2021). In a recent study, Syntelin also showed inhibition of proliferation and metastasis of triple-negative breast cancer and rarely led to cell necrosis (Mullen et al., 2021).

### 6.5 UA62784 and its derivatives

UA62784 is a novel fluorenone that specifically inhibits pancreatic cancer cell lines (Henderson et al., 2009) (Figure 5I). UA62784 inhibits microtubule-associated ATPase activity and leads to reversible cell cycle arrest and apoptosis in pancreatic carcinoma (Henderson et al., 2009). Previously, UA62784 was revealed as an inhibitor of CENP-E, and showed effective anti-tumor activity in the treatment of pancreatic cancer (Henderson et al., 2009). More than eighty UA62784 analogs have been synthesized and tested, however, there is no improvement in the selectivity pancreatic cancer of and kinesin-specific inhibitory patterns of the lead analog UA62784, excluding two analogs PC-046 and PC-053 (Shaw et al., 2009). However, Tcherniuk et al. (2011) have shown that UA62784 does not inhibit CENP-E ATPase activity but stimulates microtubule depolymerization through the interactions with microtubule near colchicine binding site using biophysical binding studies and *in vivo* imaging (Tcherniuk et al., 2011). The utilization of biophysical methods, molecular mass spectrometry imaging, live cell imaging, and optical tweezers would gain insight into the targets of small molecular compounds (Calligaris et al., 2010; Calligaris and Lafitte, 2011), which may reveal the truth and resolve disputes. The effect of UA62784 is superimposed with other microtubule targeting drugs currently used in the clinic, such as vinblastine, which makes it possible that UA62784 may be used in combination with vinblastine to avoid drug resistance of tumor cells. In addition, though UA62784 does not inhibit CENP-E, it also shows specific cytotoxicity to pancreatic cancer locus 4 (DPC4)-deficient cancer cells (Wang et al., 2009), and the mechanisms remain to be studied in the future.

### 6.6 Benzo [*d*]pyrrolo [2,1-*b*] thiazole derivatives

A new kind of CENP-E inhibitor, benzo [*d*]pyrrolo [2,1-*b*] thiazole derivatives (Figure 5J), was identified through the screening of a small-molecule chemical library (Yamane et al., 2019). This compound suppresses the microtubule-stimulated ATPase of CENPE’s motor domain with an IC_50_ of 17 μM in an ATP-competitive behavior (Yamane et al., 2019). Benzo [*d*]pyrrolo [2,1-*b*] thiazole derivatives induce cell cycle arrest, apoptosis, and the inhibition of cell proliferation in HeLa and HCT116 cells (Yamane et al., 2019).

CENP-E inhibition results in the aneuploidy-mediated p53-dependent post-mitotic apoptosis, which is different from Eg5 inhibition (Ohashi et al., 2015b). CENP-E inhibitors can suppress spindle assembly checkpoint-deficient cancers, which may expand the treatment window for the other chemotherapeutics. Previous studies have shown that radiotherapy combined with cell cycle inhibitors can enhance antitumor activity (Hauge et al., 2023). In-depth studies can focus on whether CENP-E inhibitors can be estimated as novel radiosensitizers for radiotherapy. Future studies on the *in vitro* antitumor activity of CENP-E inhibitors may also measure whether their effects on the cell cycle contribute to tumor radiation therapy. This may serve as adjuvant therapy in addition to chemotherapy. Considering that CENP-E is active in mitotic cells, the inhibitory effect of CENP-E inhibitors on undivided cells is limited, for example, CENP-E inhibitors are more likely to have low neurotoxicity (low levels of peripheral neuropathy), which has also been preliminarily confirmed in the results of clinical trials (Chung et al., 2012), and CENP-E remains important as a potential low-neurotoxicity antitumor target.

In summary, these CENP-E inhibitors provide useful backbones for future structural modifications and modeling studies. At present, there are various inhibitors of CENP-E, but the binding site of these inhibitors is very single. In the future, new inhibitors with other binding sites can be screened. In addition, animal experiments are necessary for existing inhibitors of CENP-E. Novel inhibitors may focus on improving their antitumor activity and minimizing adverse reactions *in vivo*.

## 7 Drug resistance mechanisms of CENP-E inhibitors: mutations, transporters, or expression alterations

**FIGURE 6:**
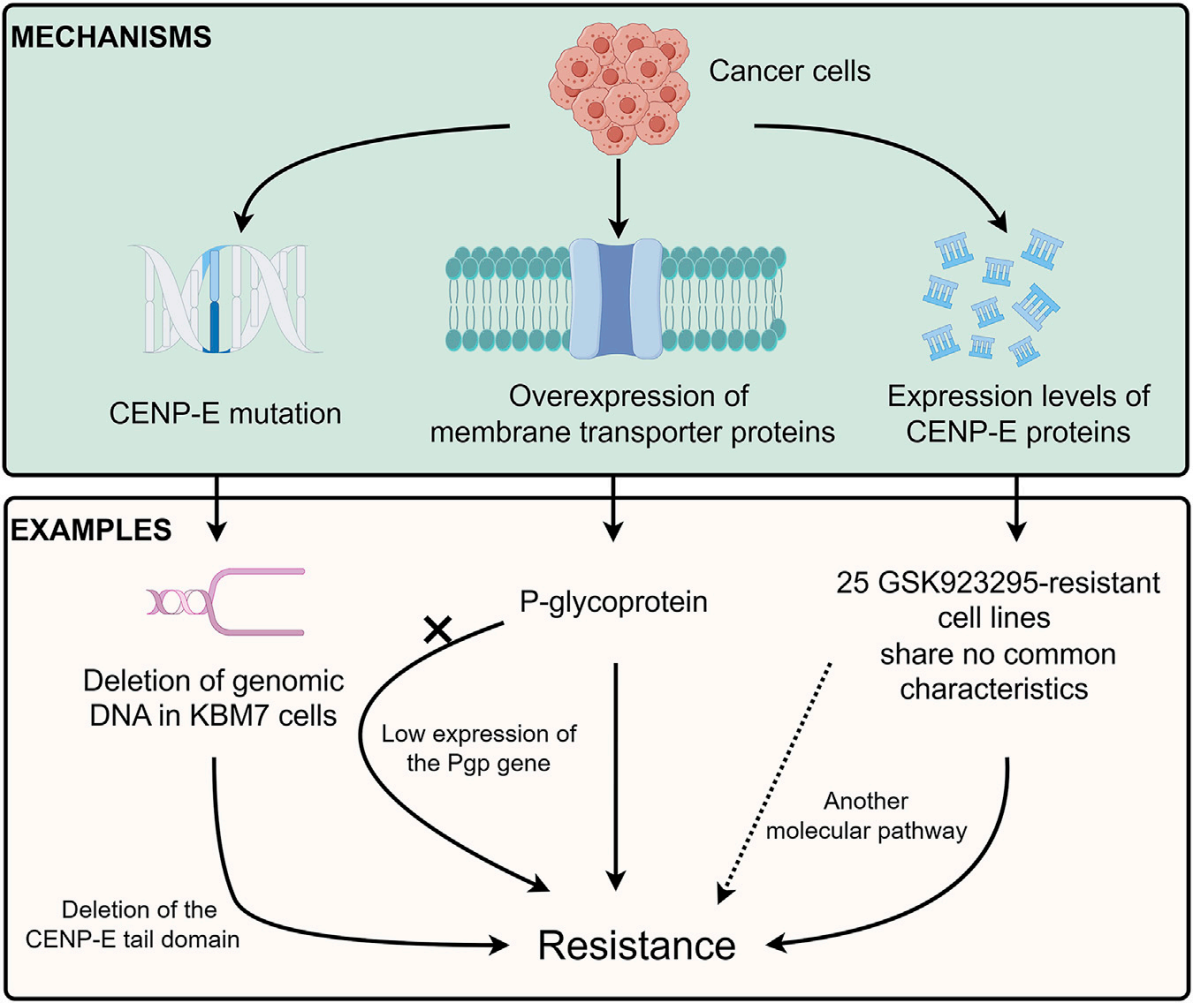
Drug resistance of CENP-E inhibitors. Tumor cells can develop resistance to CENP-E inhibitors through the *CENP-E* gene mutation, membrane transporter proteins overexpression, or their own CENP-E expression level. For example, the deletion of DNA in KBM7 cells will lead to the deletion of the CENP-E tail domain, which in turn leads to specific resistance to GSK923295. The overexpression of P-glycoprotein (Pgp) can also lead to resistance to GSK923295, but the low expression of the *Pgp* gene in GSK923295-resistant KBM7 cells suggests that cells may generate different mechanisms of drug resistance. No common characteristics of 25 GSK923295-resistant cell lines indicate that other molecular pathways lead to drug resistance.

Cancer cells usually contain chromosomal translocation, inversion, duplication, and aneuploidy, which lead to specific constraints on the evolution of genetic changes and chemotype-specific resistance (Bakhoum and Cantley, 2018). A recent study has indicated that different chromosome copy numbers in cancer cells result in distinct modes of GSK923295-specific resistance (Pisa et al., 2020) (Figure 6). The diploid HCT116 cells form the drug-specific resistance through the mutations at the GSK923295-binding site (M97V and R189M) near loop 5 of the CENP-E motor domain, which suggests that a single point mutation in CENP-E motor domain is sufficient to confer drug resistance through inhibiting GSK923295 recognition (Pisa et al., 2020). However, the near-haploid mammalian KBM7 cells show an approximately 300 kb deletion of genomic DNA, which results in the deletion of the CENP-E tail domain and GSK923295-specific resistance (Pisa et al., 2020). Together, these results suggest that distinct mechanisms of resistance can arise in cancer cells with different ploidies or karyotypes. However, how the deletion of the C-terminal domain of CENP-E increases the resistance of haploid cells to GSK923295 is unknown and remains to be revealed.

Previous studies have suggested that multidrug resistance efflux transporter P-glycoprotein (referred to as Pgp or ABCB1) is responsible for the GSK923295 resistance (Tcherniuk and Oleinikov, 2015). However, there is low or no expression of the *Pgp* gene in both parental and GSK923295-resistant KBM7 cells (Pisa et al., 2020). These results indicate that there might be different mechanisms in diverse cancer cells to generate resistance to GSK923295. In addition, according to the hints of these studies, exploring the mutations of CENP-E in drug-resistant cells may help to discover several important sites for drug binding.

Unlike multiple cancer cell lines, the SW620, CAPAN-2, and MRC5 cancer cell lines are resistant to Compound A (Ohashi et al., 2015a). These results indicate that not only low expression of CENP-E but also another molecular pathway, may be involved in the sensitivity and resistance to Compound A. Among 237 tumor cell lines, there are 25 GSK923295-resistant cell lines with no common characteristics (Wood et al., 2010). Moreover, a group of basal subtype breast cancer cells is most sensitive to GSK923295, while nonmalignant cancer cells are more resistant to GSK923295 (Wood et al., 2010). The similarities and differences between GSK923295-sensitive and non-sensitive tumor cells deserve to be studied and will help to discover the possible mechanisms of CENP-E drug resistance.

There are many determinants of sensitivity and resistance to antimitotic drugs, including the overexpression of a class of membrane transporter proteins, ABC-transporters (ATP-dependent drug efflux pumps or ATP-binding cassettes), such as the P-glycoprotein (Dumontet and Sikic, 1999; Ambudkar et al., 2003; Chanel-Vos and Giannakakou, 2010; Pote and Gacche, 2023). Moreover, cancer cells also have microtubule-related mechanisms to confer chemical resistance and generate intrinsic insensitivity to antimitotic drugs, including the expression or binding of regulatory proteins, post-translational modifications of tubulin, and abnormal expression of tubulin isotypes (Dumontet and Sikic, 1999; Gonçalves et al., 2001; Jordan and Wilson, 2004). CENP-E is related to microtubule-resistance drugs (Chanel-Vos and Giannakakou, 2010). The interactions between CENP-E and BubR1 are diminished in epothilone B-resistant A549 cells (Yang et al., 2010). In addition, whether CENP-E inhibitor-resistant tumor cells lead to cells acquiring broad-spectrum resistance to other inhibitors of mitotic kinesins deserves further exploration. Moreover, it is still unknown whether the microtubule dynamics and properties of CENP-E inhibitor-resistant cells change compared with normal tumor cells, which can be further verified by the cold treatment, the response of microtubule inhibitors colchicine, or paclitaxel.

In summary, there are three main mechanisms of drug resistance mechanisms of CENP-E inhibitors, including the gene mutations in the *CENP-E* gene (Pisa et al., 2020), the expression of the P-glycoprotein (Tcherniuk and Oleinikov, 2015), and the different expression levels of CENP-E proteins in diverse cancer cells (Wood et al., 2010) (Figure 6). These results indicate that several unidentified factors, including the overexpression of functionally redundant genes, the silence of spindle assembly checkpoints, or the resistance to cell death after chromosome instability, may contribute to different inhibitory effects of CENP-E inhibitors and drug resistance.

## 8 Conclusions and future perspectives

To date, there is a key scientific question that remains largely obscure: how does kinesin −7 *CENP-E* achieve high expression levels in a wide range of tumor tissues and cancer cells? The transcriptional regulation and intracellular environment of the CENP-E gene might be a reason for the tissue-specific expression and upregulated expression of *CENP-E*. The expression of *CENP-E* has a stable pattern in the cell cycle, but the transcription factors or regulatory proteins that regulate *CENP-E* gene expression are less studied. Thus, the in-depth studies of the promoter region, transcription factor binding site, and enhancer elements of the *CENP-E* gene will help to explain the molecular basis of *CENP-E* periodic expression and its high expression in tumors. Tumors are characterized by rapid proliferation and stronger requirements for vigorous mitosis compared with normal tissue. Thus, tumors appear to have higher expression of cell cycle regulated genes such as CENP-E, which are strictly cell cycle regulated. In particular, the low expression of CENP-E in liver cancer cells is different from the high expression in most tumors, and the underlying reasons and specific mechanisms need to be further elucidated.

The specificity and effective concentrations of the drugs on the targets are important for cancer treatment. The question of whether GSK923295 specifically targets CENP-E and its effective concentrations *in vivo*. In the future, the construct of the *CENP-E* knockout cell line using CRISPR-Cas9 gene-editing technology, together with the observations of the phenotypes and responses of CENP-E knockout cancer cells in the presence of inhibitors, would help to explore the binding specificity, off-target effects, and side effects of CENP-E inhibitors. Moreover, the differences in the effects of GSK923295 *in vivo* and *in vitro* should also take into account the following factors, including the half-life of the drugs, the metabolic pathways *in vivo*, the methods of drug administration, the target sites of action, and the cumulative concentrations of the drugs at the tumors.

At present, the development of drug combinations, including different drugs on one single target, is a new strategy to overcome drug resistance (Wylie et al., 2017; Real et al., 2020). To date, most CENP-E inhibitors bind to the motor domain and inhibit the ATPase activity of CENP-E. Therefore, the search, development, and chemical modifications of novel inhibitors targeting CENP-E’s coiled-coil or tail domain are conducive to solving the issues of drug-induced mutations in the motor domain and related drug resistances. Furthermore, a combination therapy based on CENP-E inhibitors and microtubule-targeting agents would be of high clinical advantage in wide applicability, lower toxicity, and better antitumor activity. In addition, tumor-specific targeting should also be taken into account to reduce the effects on rapidly dividing, normal cells *in vivo*.
